# A Comparative Study of the No-Punch Technique in Reducing Surgical Complications Associated with Unilateral Biportal Endoscopic Spine Surgery

**DOI:** 10.3390/jcm14207295

**Published:** 2025-10-16

**Authors:** Jwo-Luen Pao, Chun-Chien Chang

**Affiliations:** Department of Orthopedic Surgery, Far Eastern Memorial Hospital, New Taipei City 22060, Taiwan

**Keywords:** minimally invasive surgical procedure, intraoperative complications, video-assisted techniques and procedure, comparative effectiveness research, treatment outcomes

## Abstract

**Background/Objectives**: Unilateral biportal endoscopic spine surgery (UBE) has gained popularity due to its minimal invasiveness, endoscopic magnification, bloodless visual field, and broad application to various spinal disorders. We proposed the “no-punch” technique for UBE spine surgery, emphasizing its capability to prevent neural injury and preserve facet joints. This study aims to examine its efficacy in reducing the risk of incidental durotomy through a comparative study. **Methods**: A total of 914 consecutive patients with various degenerative spine disorders who underwent UBE surgery between October 2018 and July 2023 by a single surgeon in a single institute were included. The Punch Group consisted of 660 patients (830 segments) who underwent UBE surgeries using Kerrison punches. The No-Punch Group included 254 patients (330 segments) who underwent UBE surgeries without using Kerrison punches. We retrospectively reviewed the medical records and operative videos to identify surgical complications, their management, and final treatment outcomes. **Results**: Sixty-three surgical complications (58 in the Punch Group), including incidental dural tears, nerve root injuries, incomplete decompression, epidural hematoma, and broken instruments, were identified. The No-Punch Group exhibited a significantly lower overall complication rate (8.8% vs. 2.0%), along with a reduced incidence of dural tears (3.9% vs. 0) and neural injuries (5.3% vs. 0.4%). The improvement was particularly notable in lumbar decompression surgeries (5.0% vs. 0.8%) and revision surgeries (9.9% vs. 0%). **Conclusions**: The “no-punch” technique enhances the safety of UBE surgery for degenerative spine disorders by understanding the injury mechanisms and modifying the surgical techniques accordingly.

## 1. Introduction

The unilateral biportal endoscopic (UBE) technique has become one of the most important innovations in minimally invasive spine surgery over the past decade. Unlike conventional open approaches or tubular minimally invasive techniques using a tubular retractor system, UBE is performed through two independent portals without the use of retractors. This specific feature allows easy access for instruments, better ergonomics for the surgeon, and flexible maneuverability for decompression or fusion procedures. Continuous saline irrigation provides hydrostatic pressure that not only suppresses bleeding but also washes away bone debris and tissue oozing. When combined with high-definition endoscopic systems, UBE offers a bright, magnified, and nearly bloodless surgical field. These advantages allow surgeons to perform precise and delicate procedures with minimal collateral damage to the paraspinal musculature, ligaments, and facet joints.

The clinical applications of UBE have rapidly expanded. Early reports focused on lumbar discectomy for herniated discs, then on laminotomy for degenerative lumbar spinal stenosis [1,2,3]. More recently, the technique has been extended to lumbar interbody fusion for degenerative disc disease or spondylolisthesis and to posterior cervical foraminotomy for foraminal stenosis [4,5,6,7]. Across these various indications, published studies consistently report favorable outcomes, including reduced postoperative pain, shorter hospital stays, and comparable efficacy when compared with tubular minimally invasive or traditional open approaches [8,9,10,11]. These promising results have contributed to the widespread adoption of UBE in many countries.

Despite these advances, surgical complications remain a critical issue. The most common complication of UBE surgery is incidental dural tears, followed by epidural hematoma, incomplete decompression, and, less frequently, neural injury [12]. Although almost all the studies have emphasized that dural tears encountered during endoscopic surgery are usually small and can be treated with conservative measures [13,14], such conclusions may underestimate their clinical impact. Dural tears are not merely technical nuisances; they may prolong operative time, increase the risk of infection, or contribute to persistent postoperative symptoms. Importantly, surgical safety should not be justified solely by the notion of a “learning curve” [15,16,17]. While increasing surgical experience may improve outcomes, relying solely on accumulated practice cannot eliminate the risk of complications. A more proactive and reliable solution lies in identifying mechanisms of injury and modifying operative techniques accordingly.

Several articles in the literature have proposed novel techniques for repairing dural tears under endoscopic guidance. To date, the literature has focused mainly on techniques for repairing dural tears under endoscopic guidance. Several reports describe novel repair methods, including suturing, patching, and the application of fibrin sealant. However, only one investigation has emphasized strategies for preventing such complications altogether [18]. Preventive measures are more impactful, as they address the root cause of neural injuries rather than merely providing a solution once an injury has occurred.

The Kerrison punch has long been regarded as an essential tool for decompression in spine surgery. Its sharp cutting action enables the efficient removal of the lamina and ligamentum flavum. However, based on our extensive experience with over 4000 minimally invasive spine procedures, we have consistently noticed that the Kerrison punch is the instrument most frequently associated with complications, such as incidental dural tears and nerve root injuries. The risk comes from the need to insert the punch blindly beneath the lamina or ligamentum flavum before each cut, creating a moment of uncertainty where neural structures might be inadvertently injured. This risk is exceptionally high in revision surgeries, where epidural scarring and adhesion make tissue planes less clear [19].

In light of these observations, we developed the “no-punch” technique for UBE decompression. The rationale is based on two principles. First, high-speed drills and curved chisels are used to replace the Kerrison punch, allowing controlled and visualized bone removal. Second, the facet joint is undercut to create sufficient working space, enabling the removal of the ligamentum flavum either in large pieces or en bloc, along with peripheral bone fragments. This approach reduces the need for repeated blind insertions, preserves facet integrity, and ensures effective decompression. Most importantly, our experience suggests that the risk of dural tears and nerve root injuries is significantly reduced with this modification [20].

The present study was therefore designed as a comparative analysis to evaluate the effectiveness of the “no-punch” technique in UBE surgeries performed for degenerative spinal disorders. By systematically comparing complication rates, especially dural tears, between conventional UBE decompression and the no-punch approach, we aimed to determine whether modifying surgical instruments and techniques can improve patient safety. Additionally, we sought to clarify whether this strategy is applicable to extended surgical indications, including patients with more complex degenerative pathologies. The ultimate goal of this study is to contribute evidence that may guide surgical practice toward safer and more reliable minimally invasive spinal surgery.

## 2. Materials and Methods

### 2.1. Patient Selection

The study was conducted after obtaining approval from our institute’s Research Ethics Review Committee. This retrospective study recruited 914 consecutive patients with various degenerative spine disorders who underwent UBE surgery from October 2018 to July 2023. The patients were divided into the Punch Group, which included 660 former patients (830 segments) who underwent UBE surgeries using the conventional technique with Kerrison punches as the principal decompression instrument, and the No-Punch Group, which comprised 254 later patients (330 segments) who underwent UBE surgeries utilizing the “no-punch technique.”

The study included patients with refractory low back pain, radicular leg pain, single or multiple lumbar radiculopathies, and neurogenic intermittent claudication due to various degenerative lumbar spine disorders such as lumbar disc herniation, degenerative lumbar spinal stenosis, spondylolisthesis, and degenerative scoliosis. We also included patients with cervical radiculopathy resulting from disc herniation or degenerative foraminal stenosis. Patients who had undergone lumbar spine surgeries with newly developed pathology or unsatisfactory outcomes were also included. However, patients with infection, neoplasm-related pathology, or less than 6 months of follow-up were excluded. Before surgery, patients were encouraged to try conservative treatment for at least three months.

The surgical procedures were tailored to each patient based on their clinical presentation and radiological evaluation. These procedures included lumbar discectomy, lumbar canal decompression, lumbar interbody fusion, lumbar foramen decompression, posterior cervical foraminotomy, and revision surgery. All these procedures were performed using the UBE technique.

### 2.2. Surgical Techniques

**Figure 1:**
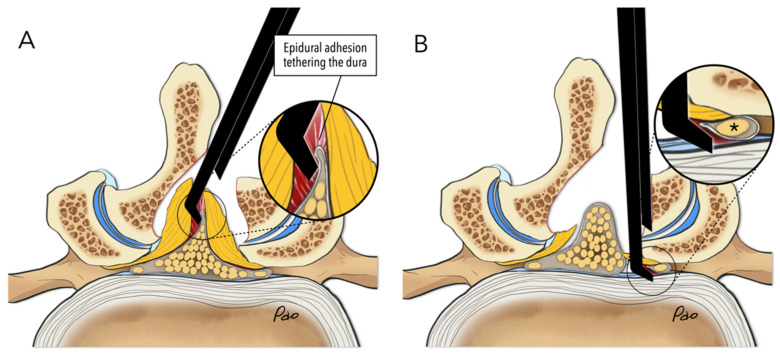
Illustrations show how neural injury occurs during the conventional decompression technique. (**A**) Blindly inserting the Kerrison punch into the central slit to remove the ligamentum flavum can cause an incidental dural tear. (**B**) Blindly using the Kerrison punch to decompress the lateral recess can cause inadvertent nerve root (the asterisk) injury.

In the Punch Group, the Kerrison punch was the primary surgical tool used to remove the lamina and excise the ligamentum flavum during UBE surgeries. The decompression technique was similar to those employed in traditional open, microscopic, or microendoscopic approaches. Decompression typically begins by drilling at the spinolaminar junction and extends cranially to expose the cranial margin of the ligamentum flavum. It then extends bilaterally to widen the surgical field. Access to the contralateral lateral recess can be achieved through sublaminar drilling after detaching the ligamentum flavum from beneath the lamina. The central slit is a longitudinal split that separates the ligamentum flavum into two halves. We are trained to insert the Kerrison punch into the central slit, cutting the ligamentum flavum into small pieces until reaching the lateral recess. If epidural adhesions beneath the ligamentum flavum are not carefully addressed, the risk of incidental dural tears increases during ligamentum flavum removal, especially when using the Kerrison punch (Figure 1A). Similarly, using the Kerrison punch to decompress the lateral recess also poses a potential risk of root injury if used blindly (Figure 1B).

**Figure 2:**
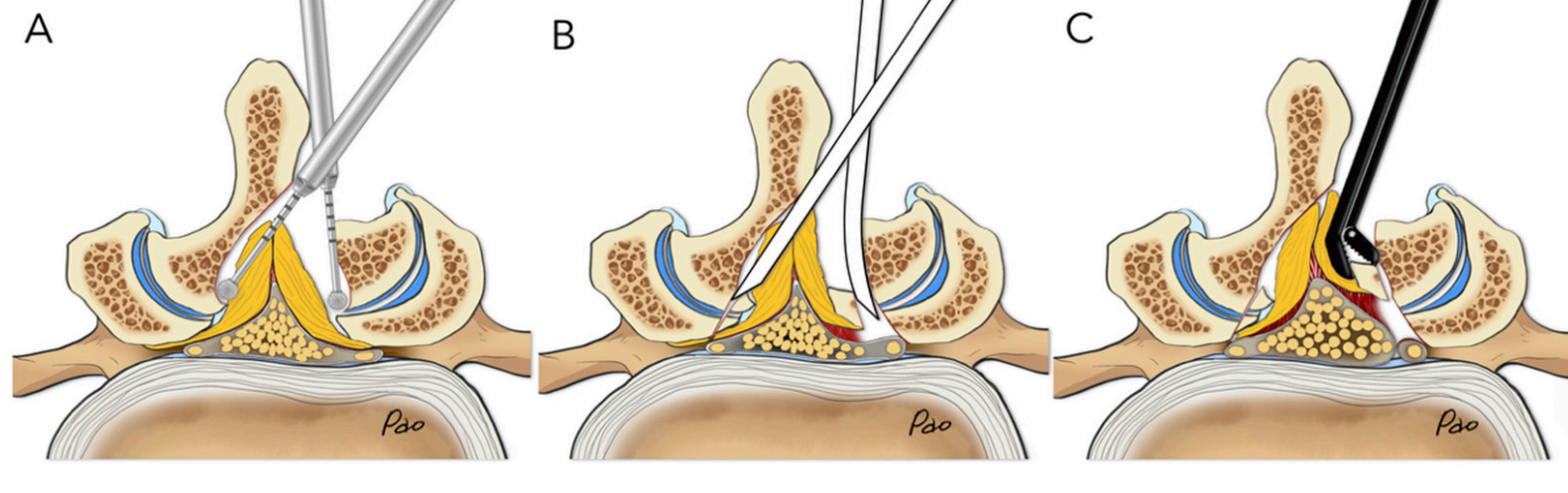
Illustrations demonstrate the “no-punch” decompression technique. (**A**) The initial laminotomy was performed with a high-speed drill featuring a coarse diamond tip. (**B**) Use straight and curved chisels to undercut the facet joint and release the ligamentum flavum from its attachment. (**C**) Utilize the micropituitary rongeur to grasp the resected lamina fragments and remove them along with the attached ligamentum flavum, either in large pieces or as a whole piece.

In the No-Punch Group, we utilize high-speed drills, curved chisels, and pituitary rongeurs as primary decompression tools to replace the Kerrison punch. We prefer using the high-speed drill with a 4 mm coarse diamond ball tip (Primado II, NSK, Tokyo, Japan) for drilling. To undercut the facet joints, we designed a set of chisels with three different curved angles: 0°, 10°, and 20°. The width of the chisel is 4 mm for lumbar spine surgery and 2 mm for thoracic and cervical spine procedures. Taking a unilateral approach for bilateral decompression in degenerative lumbar spinal stenosis from the left side as an example, we outline the surgical steps. The initial laminotomy begins by drilling through the spinolaminar junction until the cranial margin of the ligamentum flavum is exposed. The decompression continues in a counterclockwise direction to reveal the medial margin of the ipsilateral facet joint, then the caudal margin of the ligamentum flavum, and finally the medial margin of the contralateral facet joint. After detaching the ligamentum flavum from the undersurface of the lamina, we continue drilling to access the lateral recesses on both the ipsilateral and contralateral sides (Figure 2A). Straight or curved chisels are then used to undercut the facet joint and release the ligamentum flavum (Figure 2B). Straight and angled curettes are also helpful for removing any remaining ligamentum flavum attachments and separating it from its insertion site. Finally, we utilize the micropituitary rongeur to grasp the resected lamina fragments and remove them along with the attached ligamentum flavum, either in large pieces or as a whole piece (Figure 2C). Any adhesions beneath the ligamentum flavum should be released if noticed during removal. Unlike the conventional technique used in the Punch Group, we preserved the ligamentum flavum until the end of decompression, removing it in a peripheral-to-central manner.

### 2.3. Evaluation of Clinical Data and Outcomes

For analysis, demographic data, clinical information, surgical complications, and treatment outcomes were retrieved from chart review. We also reviewed all operation notes and video records to identify possible mechanisms of neural injuries, offending surgical instruments, and the management of surgical complications. All patients had a minimum follow-up of six months after the surgery.

The null hypothesis states that the “no-punch” technique does not reduce the complication rate compared to the conventional technique using Kerrison punches. The incidence of each specific surgical complication was calculated and compared between groups using the Chi-square test. The numerical data were analyzed using the Student *t*-test, with a *p*-value of <0.05 considered statistically significant.

## 3. Results

In the Punch Group, 660 patients underwent 830 segments of UBE surgeries, comprising 294 males and 366 females, with an average age of 65.4 ± 12.1 years (ranging from 20 to 92 years). In the No-Punch Group, 254 patients underwent 330 segments of UBE surgeries, comprising 111 males and 143 females, with an average age of 64.4 ± 12.0 years (ranging from 24 to 88 years). There was no significant difference in gender or age between the groups. The demographic data and distribution of each specific surgical procedure performed in each group are summarized in Table 1. There was a statistically significant difference in the distribution of surgical procedures between the Punch and No-Punch groups (*p* < 0.01). This difference likely reflects the increasing surgical maturity and confidence over time, with more complex procedures, such as lumbar interbody fusion, being performed at a higher proportion in the No-Punch Group.

There were 63 surgical complications, including incidental dural tears, nerve root injuries, incomplete decompression, epidural hematoma, and broken instruments. Of these, 26 were dural tears. Most dural tears were small and managed conservatively without direct repair. A gelatin patch covering the dural defect was usually sufficient to prevent cerebrospinal fluid leakage. Direct repair under the endoscope was performed in two patients with dural tears larger than 6 mm. Most of these patients with dural tears experienced no sequelae by the final follow-up. However, one patient had a permanent sensory deficit, while another had a permanent motor weakness.

**Figure 3:**
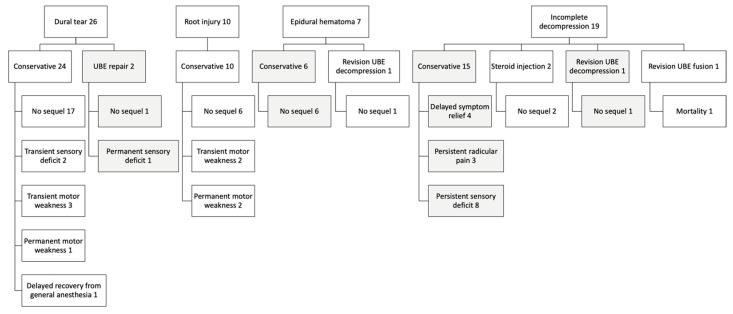
Management and outcomes of the surgical complications.

We had 10 cases of nerve root injuries, all treated conservatively, of whom two had permanent motor weakness by the final follow-up. Epidural hematomas were identified in 7 patients, with no sequelae following conservative treatment or revision UBE decompression. Nineteen patients experienced incomplete decompression; of these, 3 had persistent radicular leg pain, 8 had a permanent sensory deficit, and one died from a cardiovascular event after revision UBE fusion surgery. The management and outcomes of these complications are summarized in Figure 3.

The most common complications were dural tears, followed by incomplete decompression, nerve root injury, epidural hematoma, and broken instruments. The overall complication rate and incidence of dural tears were significantly lower in the No-Punch Group (8.8% vs. 2.0%, *p* < 0.001, and 3.9% vs. 0%, *p* = 0.001) (Table 2).

In the Punch Group, revision surgery had the highest incidence of neural injuries (9.9%, including dural tears and nerve root injuries), followed by posterior cervical foraminotomy (7.7%), lumbar decompression (5.0%), and lumbar interbody fusion (2.2%). However, the incidence of neural injuries was significantly lower in the No-Punch Group (5.3% vs. 0.4%, *p* = 0.001), especially in lumbar decompression (5.0% vs. 0.8%, *p* = 0.017) and revision surgeries (9.9% vs. 0%, *p* = 0.038). The most common surgical instrument linked to neural injury was the Kerrison punch, which caused 32 out of 35 such complications (91.4%). In the No-Punch Group, only one nerve root injury was caused by the radiofrequency wand (Table 3).

The incidence of incomplete decompression and epidural hematoma did not differ significantly between groups (Table 4).

## 4. Discussion

Minimally invasive spinal techniques have been developed over the past three decades, beginning with Foley’s introduction of microendoscopic discectomy [21]. This approach employed a tubular retractor system secured to the operating table with a flexible arm, providing visualization through either an endoscope integrated into the tube or a remote microscope [22,23]. The technique was subsequently adapted for various spinal procedures addressing diverse pathologies and demonstrated clinical outcomes comparable to those of traditional open surgery. Reported advantages included smaller incisions, reduced collateral soft tissue damage, reduced postoperative pain, and accelerated recovery [24,25,26]. However, the limited working corridor created by the tubular retractor restricted visualization and reduced surgical access to the pathology.

The advancement of endoscopy technology and surgical instruments has recently enabled most minimally invasive procedures to be performed using endoscopic techniques, further reducing the wound size and collateral damage to soft tissue. [9,11,27]. The Unilateral Biportal Endoscopic (UBE) approach is a revolutionary endoscopic technique with unique features, including a clear and magnified surgical field, the ability to handle instruments with both hands independently, a spacious working environment through a small instrument portal, and the use of familiar tools from traditional open surgery [1,2,3,4,5,28,29].

Endoscopic spine surgery is technically challenging and involves a steep learning curve [15,16,17,30,31]. This learning process is usually associated with a higher complication rate, extended operation time, and prolonged hospitalization [14]. Most studies claim that complications in endoscopic spine surgery can be managed conservatively with no severe sequelae. However, potential sequelae from these complications did happen and should not be overlooked as merely part of the learning curve [13,14,32]. Surgical complications can negatively impact treatment outcomes. In our study, 17 patients experienced permanent sensory deficits, motor weakness, or persistent radicular leg pain due to surgical complications. Preventing complications rather than addressing them after they occur is always preferable. Understanding the mechanisms of injury is essential in preventing their occurrence.

The most common complication in UBE surgery is dural tears [12,33]. In addition to the direct injury to neural tissues and the potential sequelae, dural tears in UBE surgery raise specific concerns. When a dural tear occurs, saline or air bubbles may enter the cerebrospinal fluid space, potentially causing brain injury by increasing intracranial pressure or through the air bubbles themselves [34]. Small tears can be managed using fibrin sealant or gelatin patches to prevent cerebrospinal fluid leakage. If the dural tear exceeds 10 mm, immediate repair is strongly recommended once it is identified. Large dural tears can be repaired using non-penetrating hemostatic clips or direct sutures [13,14,35,36]. However, non-penetrating hemostatic clips are not readily available in every hospital, and direct repair under the endoscope is highly technically demanding and only feasible in an expert’s hands [18]. In our study, the incidence of dural tears was 2.8%, and direct repair under the endoscope was performed in two patients. All these dural tears occurred in the Punch Group. The treatment outcomes were generally good, although one patient experienced permanent sensory deficits, another had permanent motor weakness, and one had a delayed recovery from general anesthesia.

**Figure 4:**
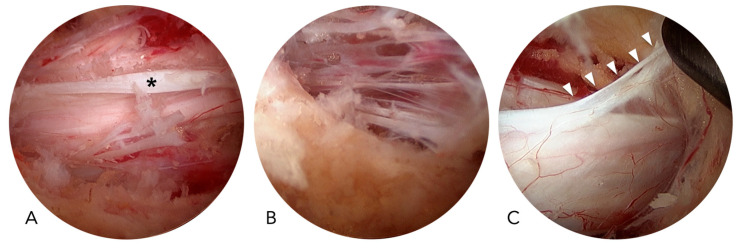
Endoscopic photos show (**A**) the central dural fold (asterisk), (**B**) the epidural adhesion in a case with severe stenosis, and (**C**) the meningovertebral ligament (white arrowheads).

In UBE surgery, dural tears are more common in the central region than in the lateral areas. Several anatomical and pathological factors, including the central dural fold, epidural adhesions, and the meningovertebral ligaments, contribute to this increased risk (Figure 4). The central dural fold is a unique phenomenon associated with endoscopic spine surgery performed with normal saline irrigation. In this setting, the central dura, which is connected to the ligamentum flavum or lamina via fibrotic tissue and covered by epidural fat, becomes tethered while the surrounding dural sac is compressed by hydrostatic pressure. This tethering produces a characteristic central fold of the dura [14]. Epidural adhesions, commonly encountered in revision surgeries, may also occur in primary cases with severe stenosis. The meningovertebral ligaments, which anchor the dura to the ligamentum flavum or lamina, pose additional risk because they can cause dural lacerations if forcefully stretched without proper caution [37,38]. All these structures are located in the central dural area and are hidden by the ligamentum flavum. Therefore, attempting to remove the ligamentum flavum through a central slit without carefully recognizing these features may lead to dural injury.

Kerrison punches are the most crucial surgical tools for neural decompression but carry significant risks of dural tears and nerve root injuries [14]. They are convenient and efficient for resecting the lamina and removing the ligamentum flavum. The ligamentum flavum acts as a natural barrier that protects the dura mater during neural decompression. Nevertheless, this structure must be excised to achieve adequate neural decompression. Traditionally, surgeons tend to insert the Kerrison punch into the central slit to remove the ligamentum flavum in a central-to-peripheral and piecemeal manner. If the central dural fold, epidural adhesion, or meningovertebral ligament is not properly identified, the dura hidden by the ligamentum flavum may be inadvertently damaged by the Kerrison punches [14,36]. A massive dural tear may occur if the surgeon continuously pulls the ligamentum flavum along with the dura. In our study, the Kerrison punch was responsible for 25 dural tears and seven nerve root injuries, which account for 91% of the total 35 neural injuries in the Punch Group. In contrast, only one nerve root injury caused by the radiofrequency wand occurred in the No-Punch Group. There is only one study in the literature reporting on modifications of surgical techniques to prevent dural tears [18]. Using their specially designed dural protectors, Hong et al. effectively prevented dural tears in the nerve root area. However, in his study, dural tears still occurred in the central thecal area.

Our “no-punch” decompression technique effectively reduces the risk of dural tears and nerve root injuries by avoiding the previously mentioned injury mechanisms. We abandoned the most dangerous surgical instrument, the Kerrison punch. We performed the decompression in a peripheral-to-central manner, releasing the ligamentum flavum from its periphery and removing the ligamentum flavum in large pieces along with attached laminotomy bone chips. All these modified techniques contribute to the very low incidence of neural injuries. The incidence of dural tears was significantly reduced from 3.9% to 0%. Additionally, the overall incidence of surgical complications was significantly reduced from 8.8% to 2.0%.

Across various surgical procedures, the overall incidence of neural injury in our series decreased significantly, from 5.3% to 0.4%, by switching from the conventional punch technique to the no-punch technique. Neural injuries are most common in revision surgeries, where epidural adhesive scarring from previous operations increases the risk of accidental dural or neural damage. Notably, in the revision subgroup of our No-Punch Group, no neural injuries were observed. This finding highlights the effectiveness of the “no-punch” technique in managing challenging cases with dense epidural adhesions.

Epidural adhesive scarring is usually most prominent in the central thecal sac, where dense fibrotic attachments and distorted anatomy increase the risk of direct neural injury. In contrast, scarring is generally less severe in the peripheral regions, where safer dissection planes are often identifiable. Our “no-punch” technique leverages this anatomical difference by using a high-speed bur to thin the residual lamina, followed by curved chisels or osteotomes to release adhesions from the peripheral margins. By avoiding aggressive central dissection and minimizing direct manipulation of the dura and nerve roots, this approach decreases the risk of dural tears or neural trauma.

This approach marks a significant shift in UBE surgery, focusing on controlled, peripheral dissection instead of central exposure. It is especially beneficial in revision procedures where the risk of nerve injury is usually higher. By enhancing safety and consistency, the “no-punch” technique can be a valuable improvement for surgeons seeking to minimize complications while maintaining effective decompression.

The observed difference in procedural distribution between groups should not be interpreted as a selection bias but rather as an evolution of surgical expertise over time. As the surgeon’s proficiency in UBE techniques improved, more complex surgeries, such as lumbar interbody fusions and multilevel decompressions, were increasingly performed in the No-Punch Group. This shift in case complexity highlights the growing confidence in the no-punch technique, not only as a safer alternative but also as a reliable method capable of addressing more technically demanding pathologies.

Future studies should include postoperative MRI analysis to measure the effectiveness of neural decompression and the degree of facet joint preservation between the conventional and “no-punch” techniques. Long-term serial imaging will be essential to monitor the development of post-decompression segmental instability, especially in cases involving significant bony or ligamentous resection. Comparing radiological indicators such as facet joint integrity, epidural space restoration, and motion preservation might provide additional insights into the biomechanical benefits of the “no-punch” technique. Furthermore, developing standardized surgical protocols and structured training programs will be essential for improving reproducibility, shortening the learning curve, and encouraging wider clinical adoption across institutions with varying levels of endoscopic expertise. These efforts will help ensure that the safety and effectiveness demonstrated in this study can be effectively translated into routine clinical practice.

This study has several limitations. First, it employs a retrospective design, which inherently limits the ability to establish causal relationships. Second, patients in the Punch and No-Punch groups were not randomly assigned; group allocation was based on the timing of the surgery, which may introduce chronological bias. The improved outcomes observed in the No-Punch Group might partly be due to the surgeon’s increasing experience with UBE techniques and greater confidence in managing more complex cases. Another significant limitation is that all surgeries were performed by a single surgeon at a single institution. While this ensured consistency in procedures and minimized inter-operator variability, it also restricts the generalizability of the findings can be applied. Additionally, the surgeon’s personal preferences and comfort with certain instruments, such as the Kerrison punch versus high-speed drills, could have influenced both the technique used and the perceived outcomes.

Despite these limitations, we believe the current study offers valuable insights into the injury mechanisms of UBE spine surgery and endorses the no-punch technique as a promising approach to reduce neural complications. Additional multicenter, prospective studies involving surgeons with different levels of experience are needed to confirm the reproducibility and wider applicability of this technique.

## 5. Conclusions

Practice helps surgeons overcome the learning curve, reduces operation time, and improves treatment outcomes. However, avoiding the same mistakes is impossible without thoroughly understanding why and how they occur. This study aims to identify the mechanisms of injury and demonstrate the “no-punch” technique as an effective surgical method for preventing neural injury and lowering the overall complication rate of UBE surgery.

## Figures and Tables

**Table 1:** Demographic data.

	Punch	No-Punch	*p*-Value
Patients	660	254	
Male	294	111	0.82 *
Female	366	143	
Mean age	65.4 ± 12.1	64.4 ± 12.0	0.42 **
Age range	20~92	24~88	
Segments	830	330	
Procedures			
Lumbar canal decompression	237 (35.9%)	73 (28.7%)	<0.01 *
Lumbar interbody fusion	137 (20.8)	87 (34.3%)	
Lumbar discectomy	116 (17.6%)	42 (16.5%)	
Lumbar foramen decompression	46 (7.0%)	16 (6.3%)	
Posterior cervical foraminotomy	13 (2.0%)	10 (3.9%)	
Revision surgery	111 (16.8%)	26 (10.2%)	

* Chi-square test; ** independent *t*-test.

**Table 2:** Comparison of complications between groups.

	All		Punch		No-Punch		*p*-Value *
	Patient	%	Patient	%	Patient	%	
Dural tears	26	2.8%	26	3.9%	0	0.0%	0.001
Nerve root injuries	10	1.1%	9	1.4%	1	0.4%	0.207
Incomplete decompression	19	2.1%	16	2.4%	3	1.2%	0.238
Epidural hematoma	7	0.8%	6	0.9%	1	0.4%	0.423
Broken instruments	1	0.1%	1	0.2%	0	0.0%	0.535
Total	63	6.9%	58	8.8%	5	2.0%	<0.001

* Chi-square test.

**Table 3:** Distribution and comparison of neural injuries and offending instruments in subcategories of surgical procedures between groups.

	Punch					No-Punch					
	Patient	Dural Tear	Root Injury	Neural Injury		Patient	Dural Tear	Root Injury	Neural Injury		
	(n)	(A)	(B)	(A + B)	%	(n)	(A)	(B)	(A + B)	%	*p*-value
**Procedures**											
Lumbar decompression *	399	13	7	20	5.0%	131	0	1	1	0.8%	0.017
Canal decompression	237	12	3	15	6.3%	73	0	1	1	1.4%	0.052
Foramen decompression	46	0	2	2	4.3%	16	0	0	0	0.0%	0.380
Discectomy	116	1	2	3	2.6%	42	0	0	0	0.0%	0.282
Lumbar interbody fusion	137	2	1	3	2.2%	87	0	0	0	0.0%	0.282
Posterior cervical foraminotomy	13	0	1	1	7.7%	10	0	0	0	0.0%	0.535
Revision surgery	111	11	0	11	9.9%	26	0	0	0	0.0%	0.038
Total	660	26	9	35	5.3%	254	0	1	1	0.4%	0.001
**Offending Instruments**											
Kerrison punch	660	25	7	32	4.8%	254	-	-	-	-	
Radiofrequency wand	660	1	2	3	0.5%	254	0	1	1	0.4%	
Pituitary rongeur	660	0	0	0	0%	254	0	0	0	0.0%	

* Lumbar decompression includes lumbar canal decompression, foramen decompression, and discectomy.

**Table 4:** Distribution and comparison of epidural hematoma and incomplete decompression in subcategories of surgical procedures between groups.

	Punch			No-Punch			
	Patient	Occurrence	%	Patient	Occurrence	%	*p*-Value *
**Incomplete decompression**							
Lumbar canal decompression	237	4	1.7%	73	0	0.0%	0.214
Lumbar foramen decompression	46	1	2.2%	16	2	4.3%	0.132
Lumbar discectomy	116	1	0.9%	42	1	0.9%	0.483
Lumbar interbody fusion	137	0	0.0%	87	0	0.0%	-
Posterior cervical foraminotomy	13	1	7.7%	10	0	0.0%	0.535
Revision surgery	111	9	8.1%	26	0	0.0%	0.061
Total	660	16	2.4%	254	3	0.5%	0.238
**Epidural hematoma**							
Lumbar canal decompression	237	2	0.8%	73	1	0.4%	0.830
Lumbar foramen decompression	46	0	0.0%	16	0	0.0%	-
Lumbar discectomy	116	1	0.9%	42	0	0.0%	0.535
Lumbar interbody fusion	137	3	2.2%	87	0	0.0%	0.282
Posterior cervical foraminotomy	13	0	0.0%	10	0	0.0%	-
Revision surgery	111	0	0.0%	26	0	0.0%	-
Total	660	6	0.9%	254	1	0.2%	0.423

* Chi-square test.

## Data Availability

The raw data supporting the conclusions of this article will be made available by the authors on request.

## Abbreviations

The following abbreviations are used in this manuscript:

UBE	unilateral biportal endoscopy
